# The Repeatability of Adaptive Radiation During Long-Term Experimental Evolution of *Escherichia coli* in a Multiple Nutrient Environment

**DOI:** 10.1371/journal.pone.0014184

**Published:** 2010-12-02

**Authors:** Gerda Saxer, Michael Doebeli, Michael Travisano

**Affiliations:** 1 Department of Biology and Biochemistry, University of Houston, Houston, Texas, United States of America; 2 Department of Zoology, University of British Columbia, Vancouver, British Columbia, Canada; 3 Department of Ecology and Evolutionary Biology, Rice University, Houston, Texas, United States of America; 4 Department of Mathematics, University of British Columbia, Vancouver, British Columbia, Canada; 5 Department of Ecology, Evolution and Behavior, University of Minnesota, St. Paul, Minnesota, United States of America; University of Ottawa, Canada

## Abstract

Adaptive radiations occur when a species diversifies into different ecological specialists due to competition for resources and trade-offs associated with the specialization. The evolutionary outcome of an instance of adaptive radiation cannot generally be predicted because chance (stochastic events) and necessity (deterministic events) contribute to the evolution of diversity. With increasing contributions of chance, the degree of parallelism among different instances of adaptive radiations and the predictability of an outcome will decrease. To assess the relative contributions of chance and necessity during adaptive radiation, we performed a selection experiment by evolving twelve independent microcosms of *Escherichia coli* for 1000 generations in an environment that contained two distinct resources. Specialization to either of these resources involves strong trade-offs in the ability to use the other resource. After selection, we measured three phenotypic traits: 1) fitness, 2) mean colony size, and 3) colony size diversity. We used fitness relative to the ancestor as a measure of adaptation to the selective environment; changes in colony size as a measure of the evolution of new resource specialists because colony size has been shown to correlate with resource specialization; and colony size diversity as a measure of the evolved ecological diversity. Resource competition led to the rapid evolution of phenotypic diversity within microcosms. Measurements of fitness, colony size, and colony size diversity within and among microcosms showed that the repeatability of adaptive radiation was high, despite the evolution of genetic variation within microcosms. Consistent with the observation of parallel evolution, we show that the relative contributions of chance are far smaller and less important than effects due to adaptation for the traits investigated. The two-resource environment imposed similar selection pressures in independent populations and promoted parallel phenotypic adaptive radiations in all independently evolved microcosms.

## Introduction

Adaptive radiations are a major source of biological diversity [1]. Prime examples of adaptive radiations include Darwin's Finches on the Galapagos Islands, Hawaiian honeycreepers [1], cichlid fishes in the great African lakes [2], and Anolis lizards in the Greater Antilles [3]. Adaptive radiations occur when empty and underutilized niches present new ecological opportunities [1], [4]. The underlying mechanisms of adaptive radiations are beguilingly simple: competition for limited resources and trade-offs in resource use result in the evolution of new resource specialists that are maintained by frequency-dependent selection. Reproductive isolation frequently evolves as a byproduct of ecological specialization. Despite the widespread occurrence and attractive mechanistic simplicity of adaptive radiations, the evolutionary outcome of an instance of adaptive radiation cannot generally be predicted with any degree of confidence. The inability to make such a prediction is due in part to an inability to evaluate the relative roles of *chance* and *necessity* [5] in promoting divergence [6].

Chance and necessity have opposing effects on the outcome of adaptive radiations, although both are clearly necessary for the *de novo* evolution of novel specialists. If chance, the random appearance and fixation of new mutations, is the dominant process, then adaptive radiations will be neither repeatable nor predictable. Different instances of adaptive radiation will yield different niche specialists and different ecological relationships among specialists, despite historical constraints or similarities among selective environments. If necessity, natural selection and adaptation through competition and trade-offs, dictates the outcome, then adaptive radiations will be largely repeatable, yielding similar niche specialists and ecological relationships. Examples of adaptive radiations suggest that either chance or adaptation can be the dominant factor in shaping the adaptive process and the resulting adaptive radiations [7], [8]. The historical nature of adaptive radiations has limited analyses to comparative approaches [3], [9], [10]. To directly test the relative contributions of chance and necessity to adaptive radiations, replication is essential, but difficult to achieve [3], [11] because it would require that there are (or were) multiple independent populations introduced into new locales conducive to adaptive radiation. However, different locales differ in environmental conditions, making it difficult to distinguish the effects of selective differences from those of stochastic processes. Similarly, initial genetic variation among populations is likely to further affect the outcome and is not easily incorporated into an analysis.

To disentangle the effects of chance and adaptation during adaptive radiations, we started a series of long-term selection experiments with the microbe *Escherichia coli* [12], [13], [14], [15], [16]. Here, we investigated the relative contributions of chance and adaptation and the potential for parallel phenotypic evolution among twelve independent populations evolved in a well-mixed environment containing two distinct resources: glucose and acetate. Glucose is the preferred carbon source of *E. coli*. Acetate is a by-product of glycolysis and is a low energy carbon source that is used through the glyoxylate-bypass when other carbon sources become scarce. Previous studies have shown strong trade-offs associated with specialization to glucose and acetate [12], [17]. The combination of these two distinct resources and trade-offs associated with resource specialization is expected to impose strong selection pressures, which should be reflected in strong contributions of necessity and a high degree of repeatability among independently evolved microcosms.

To assess the relative roles of chance and necessity during the evolution of adaptive radiations, we assessed the degree of divergence within and among populations for three phenotypic traits: fitness relative to the ancestor, mean colony size, and colony size diversity. We used relative fitness as a measure for adaptation. Our experimental conditions directly selected for increased fitness. Therefore, we expected strong contributions of adaptation and necessity and a high degree of parallelism. Colony morphology is commonly used to distinguish bacterial genotypes [18], because individual colonies are founded by a single bacterium. In addition, colony morphology has repeatedly been linked to resource specialization. Novel acetate specialists, in particular, form smaller colonies when plated on agar plates [12], [17], [19]. Therefore, we expected that mean colony size would decrease and colony size diversity would increase with increasing frequency of acetate specialists in the populations. Colony size and colony size diversity within microcosms are both correlated traits, because all microcosms evolved in well-mixed liquid medium and were never able to form colonies during the selection experiment. Therefore, we expected larger contributions of chance to the evolution of mean colony size and colony size diversity, which would result in less parallel evolution among independent microcosms.

Ecological diversity of different resource specialists is often maintained by negative frequency dependence [20], [21]. In prior work, we tested whether frequency-dependent selection could maintain different resource specialists in our derived microcosms by studying three microcosms in depth [12], [15]. Results from those studies suggest that frequency-dependent selection is maintaining evolved specialists within a population. However, the three populations differed in the equilibrium frequencies of the two different morphotypes tested [12]. To assess the degree of parallelism among the twelve independently evolved microcosms, we tested the extent of frequency-dependent selection among different morphotypes and assessed the degree of parallelism among the evolved microcosms.

## Results

**Figure 1:**
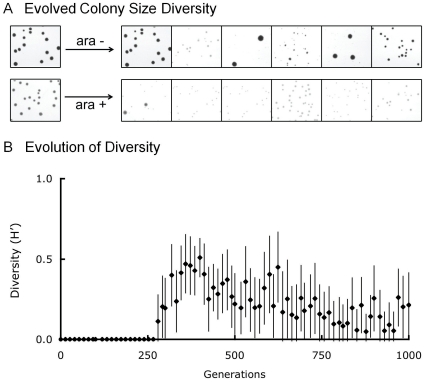
Diverse colony morphologies evolved in all twelve microcosms. (A) Colony size diversity after 1000 generations of selection. Populations are grouped by arabinose marker (ara+ and ara−), with the ancestors 606 (ara−) and 607 (ara+) to the left and the derived populations on the right. (B) Colony size diversification of the 12 microcosms. Colony size diversity (H') was calculated for every population using the Shannon-Wiener index. Each point represents the mean of colony size diversity (H') of all 12 microcosms. Error bars represent 95% confidence intervals.

Diversity in colony size evolved in all twelve microcosms propagated in medium containing a combination of acetate and glucose as the limiting resources (Figure 1A). Colony size variation was first observed at day 42 (∼280 generations) in two microcosms and in all microcosms by day 50 (∼330 generations). Colony size diversity, measured as Shannon's Index of Diversity (H'), persisted and remained relatively high throughout the selection experiment (Figure 1B).

### Relative fitness and diversity increased, while colony size decreased

To assess the trait evolution within microcosms, we assessed relative fitness, mean colony size, and colony size diversity in the ancestor and the derived populations, and performed a single factor ANOVA with a planned comparison between the ancestor and the derived populations (analyses are summarized in Table 1).

**Table 1:** Trait Evolution of Fitness, Fitness Diversity, Mean Colony Size and Colony Size Diversity in 12 Independently Evolved Microcosms after 1000 Generations (Comparison between Ancestor and Derived).

	Ancestor	Derived			Error			
Trait	Mean	Mean	MS	df	MS	df	F	*P*
Relative Fitness a	**(1)**	**1.32**	**0.106**	**1**	**6.24×10^−4^**	**11**	**168.6**	**<0.0001**
ara−	(1)	0.31	0.09	1	1.2×10^−3^	5	77.95	0.0003
ara+	(1)	0.34	0.12	1	1.4×10^−3^	5	81.87	0.0003
Fitness Variance (CV*) b	**6.73**	**7.47**	**1.53**	**1**	**5.67**	**24**	0.27	**0.61**
**Mean Colony Size (pixels)**	**121.3**	**46.7**	**1.54×10^4^**	**1**	**2.58×10^2^**	**26**	**59.91**	**<0.0001**
ara−	60.3	39.3	1.16×10^3^	1	2.70×10^2^	14	4.28	0.057
ara+	109.5	54	8.09×10^3^	1	3.43×10^2^	14	23.57	<0.001
Colony Size Diversity (CV*) c	**39.9**	**187.4**	**6.02×10^4^**	**1**	**5.38×10^3^**	**26**	**11.26**	**<0.01**
ara−	40.2	233.8	9.87×10^4^	1	6.46×10^3^	14	15.28	<0.001
ara+	39.7	140.9	2.70×10^4^	1	3.60×10^3^	14	7.49	0.011

a) Relative Fitness as determined in competition with the ancestor, by definition the relative fitness of the ancestor is 1.

b) Fitness variance determined as the CV* of relative fitness of individual colonies isolated from a microcosm.

c) Colony size diversity is assessed as CV* of colony sizes within a microcosm.

**Figure 2:**
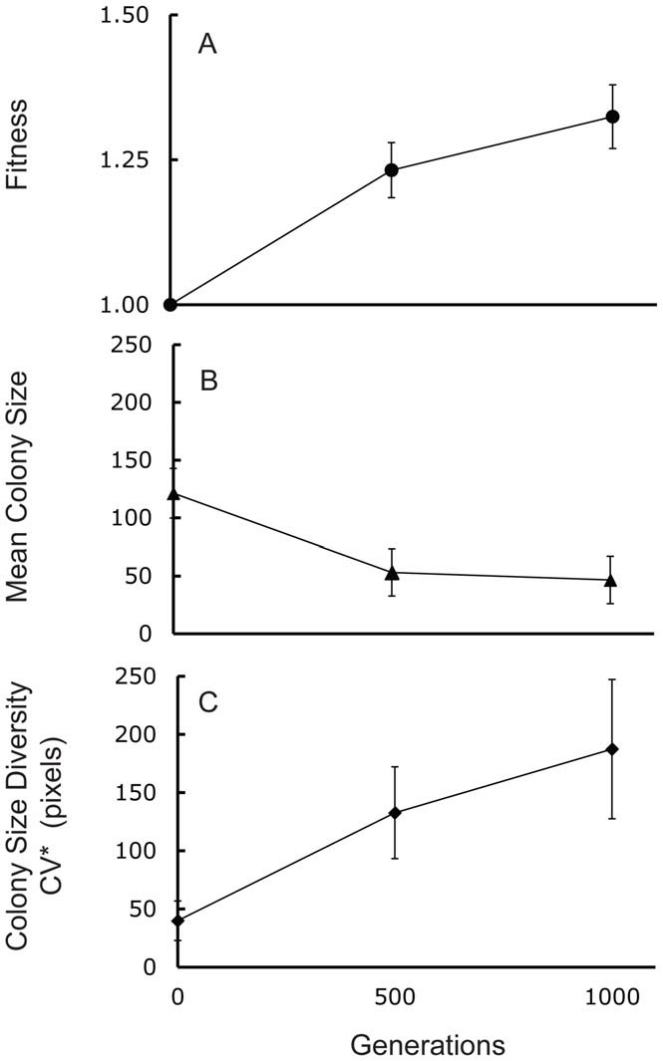
Fitness and colony size diversity increased and mean colony size decreased over the course of the experiment. Trait evolution of (A) fitness, (B) mean colony size and (C) colony size diversity at generation 500 and 1000 (mean of twelve populations with 95% CI).

Relative fitness of twelve derived microcosms had improved by 32% (Figure 2A) after 1000 generations of selection, and was significantly greater than the fitness of the ancestor with a fixed value of 1.0 (*p*<0.0001; Table 1). Relative fitness of derived microcosms against the ancestor was assayed following standard protocols [22], [23]. We used a sample of the whole microcosm in competition with the ancestor, allowing us to measure relative fitness of entire ecologically diverse microcosms. We detected no marker effect, indicating that fitness evolved similarly in ara− and ara+ microcosms (both ara− and ara+ populations: *p* = 0.0003; Table 1).

In addition to whole microcosm fitness, we also measured the fitness of individual colonies to assess the fitness differences of all readily discernible phenotypes within microcosms relative to the ancestor. Fitness variance within a microcosm was calculated as the corrected coefficient of variation, CV*, for each microcosm. Fitness variance within microcosms did not increase significantly, as determined by a comparison between ancestor and derived microcosms (*p* = 0.61; Table 1). This indicates that different colony morphs in a microcosm did not diverge in fitness, despite the evolution of ecological diversity.

To assess mean colony size and colony diversity without having to group colony sizes into discrete size categories (as is necessary for Shannon's index of diversity), we measured the size of the colonies when plated on agar plates and calculated the corrected coefficient of variation as a measure of diversity within a microcosm. Mean colony size of the microcosms plated on agar plates decreased over two-fold during the selection experiment (*p*<0.0001; Table 1; Figure 2B), which is consistent with the evolution of novel resource specialists, especially acetate specialists, which are known to form smaller colonies when plated on agar. We observed a statistically significant decrease in colony size when analyzing the ara+ populations alone, but no statistically significant decrease in the ara− populations or all populations combined (Table 1). Colony size diversity within microcosms increased over four-fold (*p*<0.01; Table 1: Figure 2C) over the course of the selection experiment. Diversity increased significantly in both, the ara− and ara+ microcosms (Table 1). The increase in colony size diversity indicates the evolution of phenotypically measurable genetic divergence within microcosms.

### The rate of evolutionary change declined

We compared the change in a trait during the first 500 generations and during the second 500 generations [22]. The rate of evolutionary change declined for all three traits (Table 2) over the course of the experiment, with statistically significant evolution over the first 500 and second 500 generations, but with a rate of change roughly two to ten times greater during generations 0–500 than during generations 501–1000. For relative fitness versus the common ancestor (*p*<0.05) and mean colony size (*p*<0.001), the declines were statistically significant, but not for colony size diversity (*p*>0.1). Colony size diversity within microcosms continued to evolve relatively rapidly, despite declining rates of evolution for mean colony size. Estimates based on variances are associated with greater uncertainties than estimates based on means. This could contribute to the non-significant decline in evolutionary change observed for colony size diversity. We did not observe a marker effect on the rate of evolutionary change.

**Table 2:** Analysis of the Rate of Change of the Microcosms over 1000 Generations.

	Rate of Change in the mean (per 1000 generations) a					
Trait	0–1000	0–500	500–1000 b	Difference c	*t_s_* d	*P* e
Relative Fitness	0.324±0.055	0.464±0.094\\	0.185±0.142	0.280±0.216	2.85	*<0.05*
Mean Colony Size f	−74.67±23.61	−136.32±43.03	−13.02±32.17	−123.3±59.51	4.55	*<0.001*
Colony Size Diversity	147.42±59.7	185.48±78.60	109.36±98.17	76.12±131.7	1.29	>0.1

a Values are means and 95% confidence intervals based upon *t*-distribution with df  = 11.

b Rate of change during the last 500 generations was calculated as twice the rate of change during 1000 generations minus the rate of change during the first 500 generations.

c Difference calculated as rate of change during the first 500 generations minus the rate of change during the last 500 generations.

d Null hypothesis is that the difference in slopes is equal to zero.

e Two-tailed probability computed from the *t*-distribution with df  = 11.

f In pixels.

### Ecological diversity within microcosms is likely maintained by frequency-dependence

**Figure 3:**
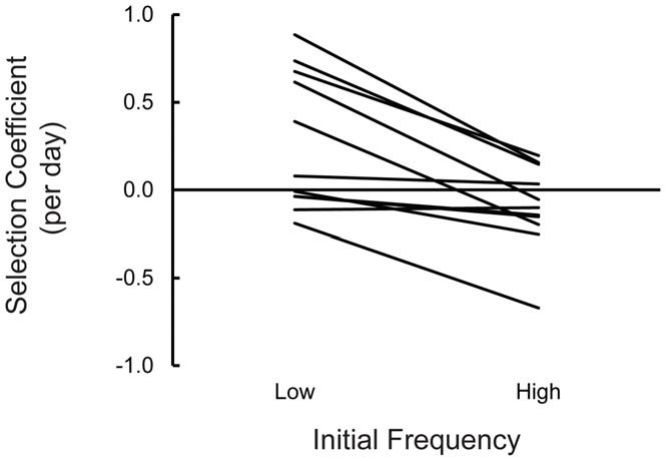
Diversity is most likely maintained by frequency-dependent selection. Genotypes have a higher selective advantage when rare (low frequency) than when common (high frequency). Selection coefficients (*s*) were calculated by linear regression of the *ln* ratio of competitor frequencies over time.

Competition experiments between pairs of the most divergent colony morphs from each microcosm indicated that frequency-dependent selection was a likely mechanism maintaining diversity within microcosms. The average difference in selection coefficients (*s*) of the small colony morph at high and low diversity over all the microcosms was 0.343 (±0.078, 95% CI), which is statistically significant by a paired-t test (*t* = 4.44, d.f. = 10, *p* = 0.0012; Figure 3). If diversity is maintained by negative frequency-dependence, the selection coefficient should be positive at low initial frequency and negative at high initial frequency. In three microcosms (#29, #33 and #36), the sign of the selection coefficient differed between low or high initial frequencies. Even in microcosms where the sign of the selection coefficients was the same at high and low initial frequencies, the small colony morphs had higher fitness when rare than when common, with the exception of two microcosms. Exclusion of microcosms #29, #33 and #36 from the analysis changed neither the magnitude of the difference (0.285±0.096, 95% CI) nor its statistical significance (paired-t test: *t* = 2.99, d.f. = 7, *p* = 0.0203), indicating that the observation of frequency-dependence is not driven simply by the most extreme examples.

The extent of frequency-dependent selection varied among microcosms. An ANOVA on the selection coefficients for the small colony morphs gives three results. First, a significant effect of initial frequency (fixed effect, *F_1,1_*_0_ = 97.21, *p*<0.0001) indicates frequency-dependent selection, as previously shown. Second, significance of microcosm (random effect, *F_10,22_* = 19.35, *p*<0.0001) indicates that the small colony phenotypes from different microcosms differed in fitness relative to their respective conspecifics, regardless of their initial frequency. Third, and most importantly, the significant interaction term (*F_10, 2_*_2_ = 4.93, *p* = 0.0009) indicates that the strength of frequency dependence varied across the microcosms. The interaction term accounted for 18.3% of the variation among measurements, approximately half of the variation associated with microcosms (39%).

### Microcosms did not diverge in fitness, but diverged in fitness variance, frequency dependence, mean colony size and colony size diversity

To assess whether the microcosms diverged for fitness, fitness variance, mean colony size, colony size diversity, and frequency dependence we performed an ANOVA and extracted the added variance component which is equivalent to the genetic variation (V_G_) among microcosms. For all traits we first performed a two-way ANOVA with microcosm and block as fixed factors. If block was not significant, we performed a single factor ANOVA. With the exception of relative fitness at generations 1000, we used a single factor ANOVA.

For fitness relative to the ancestor, we did not observe any significant genetic variation at either generation 500 or 1000 (Table 3), indicating that the microcosms did not diverge significantly in fitness at the population level. After 1000 generations however, the microcosms had diverged in fitness variance among different isolates within microcosms, which was reflected by significant genetic variation among microcosms for fitness variance among individual isolates (Table 3). Similarly, the microcosms diverged in frequency dependence between the two most divergent colony morphs, indicated by significant genetic variation among microcosms (Table 3).

**Table 3:** Genetic Variation for Relative Fitness, Fitness Variation, Frequency Dependence, Mean Colony Size and Colony Size Diversity Among 12 Independently Derived Microcosms.

Trait	Generations	Microcosms a	Block b	Error c	Var_G_	95% CI	*P*
**Relative Fitness**	500	1.7×10^−2^		8.2×10^−3^	2.90×10^−3^	0–0.0083	0.067
	1000	2.2×10^−2^	4.5×10^−3^	1.3×10^−2^	3.25×10^−3^	0–0.011	>0.1
**Fitness Variation**	1000	13.67		5.69	2.66	0–7.02	0.035
**Frequency Dependence**	1000	1.48×1		3.22×10^−2^	5.8×10^−2^	0.0078–0.132	<0.01
**Mean Colony Size (pixels)**	500	3.11×10^3^		3.07×10^2^	9.33×10^2^	409–1903	<0.0001
	1000	3.11×10^3^		2.79×10^2^	9.44×10^2^	421–1915	<0.0001
**Colony Size Diversity**	500	1.15×10^4^		2.46×10^3^	3.27×10^3^	974–10277	<0.0001
	1000	2.66×10^4^		5.81×10^3^	6.93×10^3^	2187–23650	<0.001

a Microcosms: For frequency dependence, df  = 10, all other df  = 11.

b Block: df  = 2.

c Error in Single Factor ANOVA: For relative fitness at generation 1000 df  = 22; all other df  = 24.

The microcosms also diverged in mean colony size, as indicated by significant genetic variation in mean colony size among microcosms at generations 500 and 1000 (Table 3). The minimal change in genetic variation between generation 500 and 1000 and the completely overlapping confidence intervals indicate that the populations had already diverged considerably by generation 500. A nested ANOVA indicated no significant marker effect in mean colony size at generation 500 (Marker: *F_1,10_* = 0.02, *p* = 0.87, Microcosm: *F_10,24_* = 11.08, *p*<0.0001) and generation 1000 (Marker: *F_1,10_* = 0.61, *p* = 0.45, Microcosm: *F_10,24_* = 11.57, *p*<0.0001).

Microcosms diverged significantly for colony size diversity both at generation 500 and 1000 (Table 3). Genetic diversity among microcosms for colony size diversity was already well established by generation 500, and no statistically significant change was observed after an additional 500 generations of selection. Contrary to mean colony size, a nested ANOVA revealed significant differences among the microcosms within marker as well as a significant effect of the marker both at generation 500 (Marker: *F_1,10_* = 9.41, *p* = 0.012; Microcosm: *F_10,24_* = 2.66, *p* = 0.024) and generation 1000 (Marker: *F_1,10_* = 5.67, *p* = 0.038; Microcosm: *F_10,24_* = 3.21, *p*<0.01). This marker-associated effect demonstrates the potential importance of selectively neutral genetic differences on correlated responses to selection.

### Adaptation contributed more to the evolutionary process than chance, which is reflected in parallel evolution of independent microcosms

**Figure 4:**
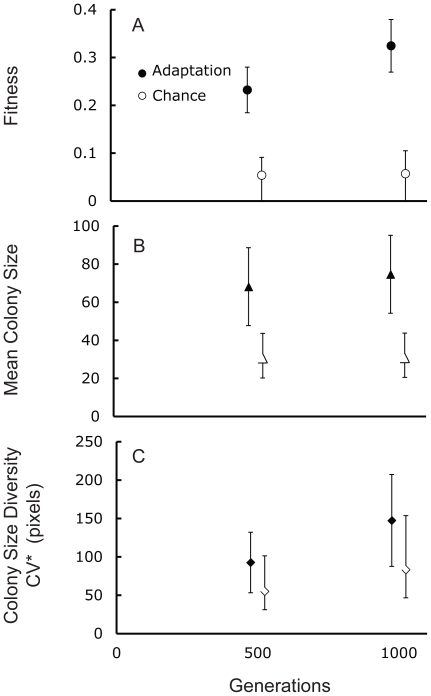
Relative contributions of adaptation were larger than chance. Relative contributions of adaptation and chance to (A) fitness, (B) mean colony size and (C) colony size diversity. Error bars represent 95% confidence intervals. The effect of adaptation was measured as the difference between the mean trait value of the derived populations and the trait value of the ancestor. The effect of chance was calculated as the square root of the genetic variation among microcosms.

To assess the relative contributions of chance and adaptation to the evolutionary process, we calculated their relative contributions. We assessed the degree of adaptation by calculating the average change in a trait [24]. To estimate the contributions of chance we calculated the square root of the genetic variation (see Material and Methods for more details). The relative contributions of adaptation were significantly larger than the contributions of chance for fitness and colony size, but not for colony size diversity, both at generations 500 and 1000, with no significant change between generations 500 and 1000 (Figure 4).

**Figure 5:**
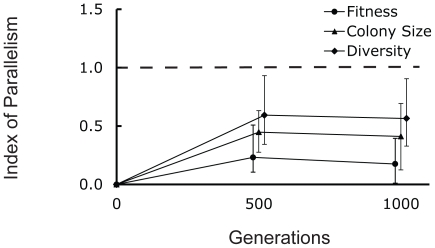
Fitness, mean colony size and colony size diversity evolved in parallel. Among microcosm divergence depicted as the index of parallelism (*I_x_*) for fitness, mean colony size and colony size diversity with confidence intervals. An index of parallelism larger than one indicates divergent evolution, while a value smaller than one indicates parallel evolution among different populations. The confidence interval was calculated by bootstrapping the data 10^6^ times.

Comparative rates of evolutionary change are difficult to interpret across experimental systems. One approach has been to measure the change in a phenotypic trait of interest over a period of time [25], which is useful when estimates of genetic change are unknown. In our microbial system, we can estimate genetic divergence without direct information on the precise genetic changes involved. To test whether the microcosms diverged or evolved in parallel, we calculated the Index of Parallelism (*I_x_*) as the ratio of effects due to chance and adaptation [26], [27]. A value of *I_x_* equal to one indicates that the differences among the microcosms are of equal magnitude to the change in the trait over the course of the selection experiment. Values less than one are indicative of greater parallelism while values larger than one indicate divergence. Despite statistically significant variation among microcosms, the microcosms evolved primarily in parallel (Figure 5) for relative fitness, mean colony size, colony size diversity and fitness variation within microcosms (*I_x_* = 0.218, CI: 0.102–0.398) as the confidence intervals (determined by bootstrapping the data 1,000,000 times) for these traits do not include one. Only frequency-dependent selection did not evolve in parallel as the confidence interval did include one (*I_x_* = 0.72, lower limit  = 0.341, upper limit  = 1.327). For relative fitness, mean colony size and colony size diversity we observe very little change in the index of parallelism between generations 500 and 1000.

## Discussion

We performed a selection experiment in a two-resource environment to assess the repeatability of different instances of adaptive radiations and the relative contributions of chance and adaptation to the evolution of fitness, mean colony size and colony size diversity. Over the course of the experiment, we observed rapid and parallel evolution of colony size diversity within microcosms. This rapid evolution of diversity is consistent with the general pattern of adaptive radiation, which is characterized by fast divergence followed by long-term persistence of diversity [1]. As we expected, we observed significant increases in relative fitness and colony size diversity and a significant decrease in mean colony size, indicating that the microcosms adapted to the selective environment and evolved different resource specialists forming smaller colonies. All three traits showed larger contributions of adaptation than chance, and evolved in parallel in independent microcosms, despite the evolution of diversity within microcosms. The consistent evolution of diversity in independent microcosms is notable, considering that any diversity within microcosms evolved *de novo* via mutations arising during the course of selection in a relatively simple, well mixed, two-resource environment, and indicates that diversification within microcosms was itself adaptive.

### The evolution of ecological diversity

The underlying causes of adaptive radiations are competition for limited resources that lead to resource specialization, and trade-offs associated with resource specialization [1], [4], [21]. The selective environment contained two distinct carbon sources, acetate and glucose, for which strong trade-offs associated with resource specialization have been observed [12], [17]. Colony size diversity evolved rapidly in all microcosms indicating the evolution of different resource specialist. Colony size is a heritable trait (see Material and Methods; [13], [17], [19], [28] that has frequently been correlated with quantitative differences in nutrient use in other experiments with the same and other ancestral genotypes [12], [13], [17], [19], [29]. Nonetheless, variation in colony size could simply have evolved due to accumulation of mutations having no importance in the selective environment [30]. This would seem particularly plausible in this study, as we evolved the populations in liquid, well-mixed, minimal nutrient environments and assessed colony size on nutrient rich agar. However, since we worked with large populations, such an accumulation of mutations due to drift is highly unlikely. Indeed, in depth analysis of the nutrient specialization of very distinct small and large colony morphs of three populations provided strong support for resource specialization [12]. While both colony types exhibited diauxic growth, during which they first utilize glucose through glycolysis before using acetate through the TCA cycle, the small and large colony morphs differed in their ability to switch between the use of glucose and acetate, with the small colonies being able to switch much faster. Similarly, Tyerman et al. [31] were able to show that competition for resources lead to ecological character displacement and the evolution of a small morphed acetate specialist (fast switcher) and a larger morphed glucose specialist (slow switcher).

The resource specialists are most likely maintained by frequency dependence (our data and [12], [15]). We observed strong negative frequency dependence in three of the microcosms. This variation in the strength of frequency dependence could be due to the fact that we only assayed the two phenotypically most divergent morphs. More extensive sampling of different colony morphs in different pair-wise combinations, or competition of the different colony morphs against the source population at different initial frequencies, may have led to a better support of negative frequency dependence. Both frequency dependence, a measure of the strength of niche specificity, and colony size, a correlated trait without direct benefits and cost, indicated that ecological diversity within microcosms evolved over the course of the selection experiment. We can compare both measurements of ecological diversity – colony size diversity and frequency-dependent selection – by comparing the coefficients of variation for both traits over the eleven microcosms assayed for frequency dependence. The corrected coefficient of variation for frequency dependence was determined using the variance in selection coefficients at high and low densities, over the average selective coefficient, corrected for the small sample size. Although the average coefficient of variation for frequency dependence is larger than for colony size (268.38 versus 170.19), a paired t-test did not indicate a statistical difference between the two measures of ecological diversity (*t* = 0.90, d.f. = 10, *p* = 0.38). This suggests that colony morphology is a good indicator of ecological diversity. In addition, small colony morphs of different microcosms have been shown to have similar ecological functions [15]. When we mixed small colonies of one microcosm with large colonies of another microcosm, the two colony morphs reached equilibrium frequencies similar to the frequencies reached by co-evolved small and large colony morphs, as long as the microcosms evolved in the same selective environment [15].

Our approach of using mean colony size as a measure of the evolved ecological diversity results in an imperfect estimate of the evolved genetic diversity, but it allowed us to capture the evolved ecological diversity, as different colony morphologies are indicative of resource specialization and ecological function. However, we are very likely to underestimate genetic diversity, as evidence suggests that different genotypes result in the same phenotype [16].

### Parallel evolution of adaptive radiations

We observed parallel evolution for all traits, except for frequency dependence. Different factors can affect parallelism and divergence. At low ecological complexity, the fitness landscape is a useful metaphor [32]. If there are no epistatic incompatibilities among beneficial mutations, and if there is only one most fit genotype out of all possible genotypes, parallel evolution is likely to occur [22], [23]. Epistatic interactions, increased environmental complexity [33], and ecological interactions can contribute to a more rugged landscape. The more rugged a landscape, the less likely it becomes that independent populations evolve in parallel. Divergence on the fitness landscape is driven by the effects of chance and contingency, and is possible even for initially isogenic populations under the same selective conditions [34]. Chance affects the occurrence and fixation of beneficial mutations, as even beneficial mutations can be lost by drift when they first appear, and are very rare [35]. Contingency can promote divergence if a fitness landscape is complex, with multiple peaks and flat areas where chance can lead to sustained divergence among populations, since subsequent adaptation is contingent on the location of each population on the landscape. The observed parallel evolution suggests relatively simple landscapes, although the divergence among microcosms suggests that there are several peaks or perhaps an adaptive ridge [36]. Even so, we cannot exclude the possibility that selection would ultimately lead to complete phenotypic convergence given sufficient time [23], [37].

Parallel evolution among independent populations has previously been observed for traits such as fitness and cell size in less complex environments [24], [27], [38]. Similar to those studies, we observed very strong parallel evolution for fitness [24], [27], despite the more complex selective environment. The predominance of parallelism in our experiment is likely due to the presence of the two nutrients used in our study, glucose and acetate, which differ in transport into cells, catabolism, and regulation. *E. coli* grows preferentially on glucose, with a regulatory structure that prevents production of proteins required for transport and catabolism of other nutrients, particularly for low energy nutrients such as acetate. These metabolic limitations can cause trade-offs for resource use [17], a requirement for the evolution of diversity through adaptive radiations [1], [21], [39]. These strong trade-offs very likely contributed to the parallel evolution among the microcosms. Such trade-offs are likely smaller in environments with two or more nutrient resources that share the same mechanisms of transport, catabolism and/or regulation because adaptation to one nutrient also confers adaptation to other resources [40]. Relaxing the trade-offs associated with resource specialization would likely reduce parallelism for correlated phenotypic characters, as the constraints on their evolution would also be relaxed. MacLean and Bell [30] observed more divergence for correlated traits when they assessed the growth of *Pseudomonas fluorescens* populations evolved in an environment with one limiting resource, and subsequently tested the correlated response in almost 100 alternate environments. The observed divergence suggests that many of these alternate environments did not involve strong trade-offs. We observed parallel evolution for the two correlated phenotypic traits, colony size and colony size diversity. However, both correlated traits are affected by nutrient specific adaptation [12], [13], [17], [29]. Our expectation is that the degree of parallelism would decrease for traits less tightly associated with fitness [27], [40] because they would experience relaxed selection and therefore accumulate random mutations. Alternatively, pleiotropic effects of mutations can lead to increased diversification of non-essential traits [41]. Therefore, if we were to extend our analysis to traits not associated with nutrient metabolism and growth, we would most likely observe less parallel evolution.

The observed parallel evolution at the phenotypic level could be due to parallel evolution at the genetic level, or due to a different combination of mutations that result in similar phenotypes. Investigation of the underlying genetic changes associated with acetate specialization indicated that the overall phenotypic similarity was not maintained at the genetic level. In one of the microcosms with strong frequency-dependent selection, Spencer et al. [16] identified a mutation associated with the physiological switch between glucose- and acetate-based growth [18]. A transposon insertion in the *iclR* gene causes de-repression of the *aceBAK* operon leading to its expression during growth on glucose, when the operon is typically repressed. As a result, genotypes with this mutation express a gene critical for acetate metabolism, malate synthase A, even during growth on glucose when this gene is normally down regulated. Genotypes with this mutation are superior competitors on acetate, but are inferior to derived glucose specialists when only glucose is available. This mutation was not found in any of the other microcosms that showed strong frequency dependence. Across microcosms, ecologically equivalent specialists arise by different mutations having the same or similar physiological impact. The equivalent specialists among microcosms remain divergent at the genetic level since there is no additional fitness benefit to fixing additional mutations having the same consequences, as they are already ecologically equivalent [42]. This pattern of overall phenotypic similarity, but divergent underlying mechanisms, is consistent with diminishing returns epistasis [6], [43]. In sexually reproducing species, this mode of divergence could lead to Dobzhansky-Muller postzygotic reproductive incompatibilities[44].

While repeated evolution of phenotypic diversity has previously been observed in more complex environments, the repeatability of the evolution of diversity in this system has not been addressed in a systematic way under the same selective conditions. For example, the rapid evolution of phenotypically distinct ecotypes in static *Pseudomonas fluorescens* microcosms has been observed repeatedly in independent experiments [45], [46], [47]. Genetic analysis showed that mutations in one of three regulatory modules can readily lead to the evolution of one of these ecotypes [48], suggesting that chance may not have a big effect on the evolution of this ecotype since other mutational pathways are far less likely. MacLean and Bell [30], [49] tested the ability of *P. fluorescens* to adapt to an array of different carbon sources and observed overall similar adaptation to the selective carbon source and more divergence in growth on other carbon sources. In our study, we quantify the parallelism during adaptive radiation in a two-resource environment by measuring multiple traits and focusing on the interactions among diverging ecological specialists. By allowing diversity to evolve within a microcosm, we select for the ability of a specialist to grow better on a particular resource, while interacting with other resource specialists during the adaptive process. These interactions can be crucial for the maintenance of the evolved diversity as shown in a different study, where diversity declined rapidly when the imposition of spatial structure interrupted established interactions among coevolved specialists [13]. Although those populations evolved in a slightly different selective environment, the observed negative frequency-dependent selection suggests that the populations also evolved important interactions in a two-resource environment. Furthermore, the experimental replication allowed us to directly track the evolution of genetic variation and to compare the degree of parallelism for different traits.

Parallel adaptive radiations are thought to be the result of similar selection pressures [3], [9], [10]. Our microcosms were exposed to identical environments. Therefore the high degree of phenotypic parallelism among our populations may not be surprising. Despite the identical selective environments and the parallelism observed for almost all traits examined we observed divergence among the populations. The partial genetic results [16] indicated larger underlying genetic variation, suggesting that not all populations evolved similarly. While the experimentally supplied resources are identical across microcosms, the evolution of resource specialists critically depends on the genetic and ecological diversity present in the population. For example, the order with which mutations arise in independent microcosms can potentially affect the fixation of subsequent mutations [50]. Our observation of diversity in frequency-dependent selection across microcosms suggests that different interactions among the ecologically diverged phenotypes evolved despite the observed parallelism at the phenotypic level.

### Conclusions

This is the first study that systematically tests the repeatability of the evolution of diversity through adaptive radiations in an environment containing two distinct resources. We observed very parallel evolution for different phenotypic traits despite divergence among independent microcosms. This suggests strong selection for certain phenotypes despite the evolution of ecological diversity and complexity. As in other studies, all diversity evolved *de novo.* However, strong trade-offs associated with nutrient specialization and a partial analysis of the underlying genetic mutations [16] suggest less parallel evolution at the genetic level and the potential for a greater number of beneficial mutational pathways than observed in other systems [48]. These results suggest that the evolution of ecological diversity through adaptive radiations can be both robust and yet surprisingly subtle in their effects.

## Materials and Methods

### Selection Experiment

We initiated and propagated the microcosms as described previously [12]. Briefly, we started the populations from two isogenic clones of *Escherichia coli* B [22], which only differed in a selectively neutral marker (ara+ and ara−) [40], [51]. We propagated the populations for 1000 generations by daily transfer of a 100-fold dilution into fresh 10 ml of Davis Minimal medium supplemented with 205 µg/ml of both acetate and glucose as two distinct limiting carbon sources. Every second day, we sampled each microcosm onto tetrazolium-arabinose (TA) agar plates, to check for cross-contamination, census each microcosm, and assess colony morphology. Every 15 days, we stored samples from each microcosm at −80°C. We isolated single genotypes from frozen microcosm samples by plating stationary phase cultures onto agar plates, and randomly selecting colonies that varied in colony size or morphology.

### Competitive Ability

Competition experiments were performed as head-to-head experiments between two competitors at different ratios (1∶10, 1∶1 or 10∶1) under selective conditions as previously described [22]. Competitors were of two types, either single genotypes or large population samples from microcosms. We estimated relative fitness of the evolved populations or single genotypes in competition with either a conspecific having a distinct colony phenotype or the reciprocally marked ancestor. Experiments with large population samples from microcosms allow estimation of the mean fitness and fitness variation that takes into account the genetic diversity within microcosms. We determined the relative fitness of the two competitors as the ratio of the number of doublings for two competitors over one day of growth [22]. A fitness of 1 indicates that both competitors are equally fit.

Fitness diversity within microcosms after 1000 generations of selection was assessed with two to six isolates obtained from each microcosm, corresponding to all the readily differentiable colony phenotypes within each microcosm, for a total of 42 derived isolates. Each isolate was competed against the alternate marker variant of the common ancestor. For each microcosm, we determined the genetic variation for fitness among the single colony isolates as the variance in fitness among all single isolates from one microcosm. We used the genetic variation to assess fitness diversity by calculating the corrected coefficient of variation
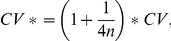
where *n* is the number of colonies analyzed and
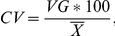
where ∑*X* is the mean trait value, in this case fitness of the colonies analyzed, and *V_G_* is the genetic variation in fitness calculated as the variance of three measurements per colony type [52]. With three-fold replication, a total of 39 measures of diversity were obtained, 36 for the derived microcosms and 3 for the ancestral marker variants.

To test for frequency-dependent selection within microcosms, we isolated two genotypes forming different colony morphologies from each microcosm after 1000 generations of selection and competed them against one another at initial frequencies of 1∶10 and 10∶1 over four (block 1) and three (block 2) days of daily transfers into fresh medium, with two replicates at each frequency. Microcosm 25 was excluded from the analyses, as we were unable to consistently distinguish the genotypes based on colony morphology. For each microcosm and block, we calculated the selection rate coefficient per day (s) of the small colony morph as the linear regression of the natural logarithm of the ratio of both competitors over time [53]. The final selection coefficient per population was calculated as the average of the two blocks.

### Colony Size and Diversity

We used two different measures of colony size. During propagation, we monitored the evolution of colony size diversity within microcosms by assessing the colony sizes of different genotypes from each microcosm every second day. Colony size classes were determined relative to other phenotypes from the same microcosm at each sample time and recorded as small, medium or large. We used the frequencies of these different colony morphologies in a microcosm to calculate Shannon's Index of Diversity (H').

To obtain a more accurate measure of mean colony size and diversity within a microcosm at generation 500 and 1000, we took digital measurements of colony sizes. Genotypes or microcosms were sampled onto TA agar plates and incubated at 37°C for 48 hours, after which we took a digital image of the plates. These images were used to measure colony sizes with Scion Image for Windows (Version Beta 4.0.2, Scion Corp, 2000). To assess colony size diversity within a microcosm, we used the corrected coefficient of variation, CV* [52]. The benefits of using CV* are that diversity can be measured without ad hoc grouping by the investigator (as would be necessary when using Shannon's Index of Diversity), variation can be assessed across populations independent of their means, and genetic variance among microcosms for CV* can be readily computed by ANOVA on the CV*. We sampled each derived microcosm and ancestral genotype onto TA agar plates, for 42 estimates for each trait at each time point.

Before using colony morphology as a trait for resource specialization, we tested the heritability of colony morphology by isolating colonies with different colony morphologies and measuring their colony size on plates before and after growth in liquid medium. The correlation coefficient between the first and second samplings of colony size of 41 isolates was 0.95 (*t* = 20.052, d.f. = 39, *p*<0.0001). This high coefficient, observed despite the single cell bottleneck and the approximately seven generations of growth in liquid medium between the two measurements, is indicative of a heritable, genetic basis for colony size.

### Statistical considerations for within microcosm evolution

To assess the changes in fitness, fitness diversity, mean colony size and diversity within microcosms between the common ancestor and the derived microcosms, we performed a one-factor ANOVA with the difference between derived and ancestral values as a planned comparison. This method is equivalent to a fixed-effects ANOVA assessing experimental treatments relative to a control and is valid given that the control is the ancestral genotype and not a single, randomly collected individual. Since relative fitness measurements are made by direct competition of the derived and ancestral competitors, a *t*-test would be an appropriate statistical test. To maintain a consistent presentation, the results can be equivalently analyzed as an ANOVA with one degree of freedom in the numerator and were presented as such. To test for a marker effect, we performed a planned comparison between the ancestor and the derived population for each marker individually.

Changes in the rate of adaptation were measured by comparing the rate of evolutionary change during the first 500 generations of selection to that during the second 500 generations. For the first 500 generations, rates of evolutionary change were obtained for each microcosm by calculating the slope between 0 and 500 generation values. The rates for the second 500 generations were obtained by substraction of the first 500 generation rate from twice the rate for the entire 1000 generations [22], [26]. Rates over all 1000 generations were calculated by determining the least squares best fit straight line anchored at the 0 generation value and through the 500 and 1000 generation values.

### Among Microcosm Divergence

To assess divergence among microcosms, we calculated the genetic variation among microcosms as the added variance component [24] by performing a two-way ANOVA, with microcosms and block as fixed factors. If we detected no significant effect of block, we performed a single factor ANOVA. Genetic variation, *V_G_* was calculated as:
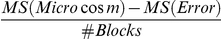

[52]. Significance of genetic variation was determined by the significance of the microcosm effect. Confidence intervals for genetic variation were calculated following the Moriguti-Bulmer procedure [52]. Marker effects among the populations were calculated by a nested ANOVA with marker and microcosms nested within marker as fixed factors for each time point and trait individually.

### Adaptation and Chance

To test for the relative contributions of chance and adaptation, we assessed the effect of chance as the square root of the genetic variation so that the effects of chance and adaptation would be of the same units. Confidence intervals were calculated as the square root of the upper and lower limits of the added variance components [52]. We measured adaptation as the difference in a trait between the ancestral value and the average value in this trait among all the derived populations. The confidence intervals were calculated as the 95% CI.

### Index of Parallelism

To calculate the index of parallelism [26], [27], we divided the square root of the genetic variation (*V_G_*(*X*)) by the adaptation measured as the average change in the trait X (Δ*X)*:
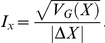



A value of smaller than one indicates parallel evolution among initially isogenic populations, while a value larger than one indicates divergent evolution. The confidence intervals were calculated by bootstrapping the data 10^6^ times.
